# Role of LECT2 in exacerbating atopic dermatitis: insight from *in vivo* and *in vitro* models via NF-κB signaling pathway

**DOI:** 10.3389/fimmu.2024.1439367

**Published:** 2024-08-14

**Authors:** Zhifang Liu, Xinyu Jiang, Keyu Zhao, Hongyu Ruan, Yizhao Ma, Yuhan Ma, Qiongyan Zhou, Jing Zhang, Xiaoyan Sun, Wenxue Ma, Suling Xu

**Affiliations:** ^1^ Department of Dermatology, The First Affiliated Hospital of Ningbo University, Ningbo, Zhejiang, China; ^2^ Health Science Center, Ningbo University, Ningbo, Zhejiang, China; ^3^ Department of Medicine, Sanford Stem Cell Institute, and Moores Cancer Center, University of California, San Diego, La Jolla, CA, United States

**Keywords:** LECT2, atopic dermatitis, NF-κB signaling pathway, inflammatory cytokines, skin barrier proteins, therapeutic target

## Abstract

Leukocyte cell-derived chemotaxin 2 (LECT2) is linked to various immune diseases. Previously, we reported that serum LECT2 levels correlate with disease severity in atopic dermatitis (AD) patients. To investigate the role of LECT2 in AD and elucidate its potential mechanisms, we used LECT2 to treat an AD mouse model induced by 1-Chloro-2,4-dinitrobenzene (DNCB) in LECT2 knockout (KO) and wild-type (WT) mice, and an AD cell model using TNF-α/IFN-γ-induced HaCaT cells. Inflammatory factors and barrier proteins were analyzed by histology, immunohistochemistry, RT-qPCR, ELISA, and Western Blot. Activation of the NF-κB signaling pathway was evaluated by Western Blot and immunofluorescence. In the AD mouse model, LECT2 treatment increased epidermal and dermal thickness, mast cell infiltration, and downregulated barrier proteins. Inflammatory factors were increased in skin lesions and serum. In the AD cell model, LECT2 decreased barrier protein levels and increased inflammatory factor levels, enhancing NF-κB P65 nuclear translocation. These results indicate that LECT2 exacerbates AD-like responses by dysregulating the NF-κB signaling pathway, highlighting its potential as a therapeutic target for AD management.

## 1 Introduction

Atopic dermatitis (AD) is a common chronic inflammatory skin disease characterized by dry skin, eczema-like skin lesions, and pruritus, often accompanied by elevated serum Immunoglobulin E (IgE) levels and inflammatory factors (1). In recent decades, the rapid development of urbanization and industrialization has significantly changed people’s lifestyles and living environments. Consequently, the incidence of AD has been on the rise, now affecting up to 30% of children and 7-14% of adults (2, 3). AD often represents the initial stage of the atopic march, potentially leading to other allergic conditions such as food allergies, asthma, and allergic rhinitis, alongside psychosocial impacts due to the skin lesions (4, 5).

The etiology and pathogenesis of AD are complex, involving skin barrier dysfunction and immune system dysfunction caused by immune, environmental, genetic, and microbial factors (3). Barrier proteins such as Filaggrin (FLG), Involucrin (IVL), and Loricrin (LOR) are crucial for maintaining skin integrity. Inflammatory responses, environmental pollution, genetic mutations, and microbial community changes can affect the expression of these barrier proteins, leading to a compromised skin barrier (6, 7). The compromised skin barrier results in dryness, allergen leakage, and microbial imbalance, triggering the immune system and further immune dysfunction (8).

Immune dysfunction in AD is mainly characterized by an imbalance between T-helper (Th)1 and Th2 cell-mediated responses (9). Th2 cells dominate in the early stage of the disease, producing cytokines like interleukin (IL)-4, IL-6, and IL-13 (10), which stimulate B cells to secrete IgE and induce keratinocytes to produce cytokines such as tumor necrosis factor-α (TNF-α), interferon-γ (IFN-γ), and regulated upon activation normal T cell expressed and secreted (RANTES). These cytokines recruit immune cells, such as T cells and eosinophils, to the site of inflammation, exacerbating the body’s inflammatory response (3, 11–13). IL-4 and IL-13 can downregulate the expression of barrier proteins such as FLG, IVL, and LOR in the skin and, together with Thymic stromal lymphopoietin (TSLP), contribute to itching (7, 14–19). IgE binds to the FcϵRI receptor on mast cells, leading to degranulation and the release of inflammatory mediators, like histamine, triggering itching and allergic reactions (20, 21). The downregulation of barrier protein expression due to immune dysfunction, coupled with skin damage caused by “itch signaling scratching”, further impairs the skin barrier, creating a “vicious circle” of worsening symptoms and barrier dysfunction.

Leukocyte cell-derived chemotaxin 2 (LECT2), primarily synthesized and secreted by hepatocytes, is a pleiotropic protein with chemotactic properties and diverse functions in various physiological and pathological abnormalities (22, 23). In bacterial sepsis, LECT2 activates Raf-1 and nuclear factor kappa B (NF-κB), enhancing macrophage phagocytosis (24, 25). LECT2 also promotes the differentiation of bone marrow-derived dendritic cells into dendritic cells, enhancing antimicrobial activity through the expression of inflammatory factors such as IL-10 and IL-23 (26). In non-alcoholic steatohepatitis (NASH), LECT2 promotes of TNF-α and Monocyte Chemoattractant Protein-1 (MCP-1) expression, increasing macrophage infiltration, and facilitating liver inflammation (27–29). In atherosclerosis (AS), LECT2 is highly expressed in diseased tissue (30) and promotes phosphorylation of c-Jun N-terminal kinase (JNK) and the expression of intercellular cell adhesion molecule-1, inflammatory factors TNF-α, IL-1β, and MCP-1, inducing an inflammatory response (31).

A previous study from our group found significantly elevated serum LECT2 in AD patients, positively correlating with the serum IgE, eosinophil levels, and disease severity (32). This study aimed to investigate the role and mechanisms of LECT2 in exacerbating AD-like responses through the NF-κB signaling pathway. We used a 1-Chloro-2,4-dinitrobenzene (DNCB)-induced AD mouse model with LECT2 knockout (KO) and wild-type (WT) mice, and a TNF-α/IFN-γ-induced human immortalized keratinocytes (HaCaT) cell model to simulate AD condition.

## 2 Results

### 2.1 LECT2 exacerbates the skin manifestations in DNCB-induced AD-like skin lesions

**Figure 1:**
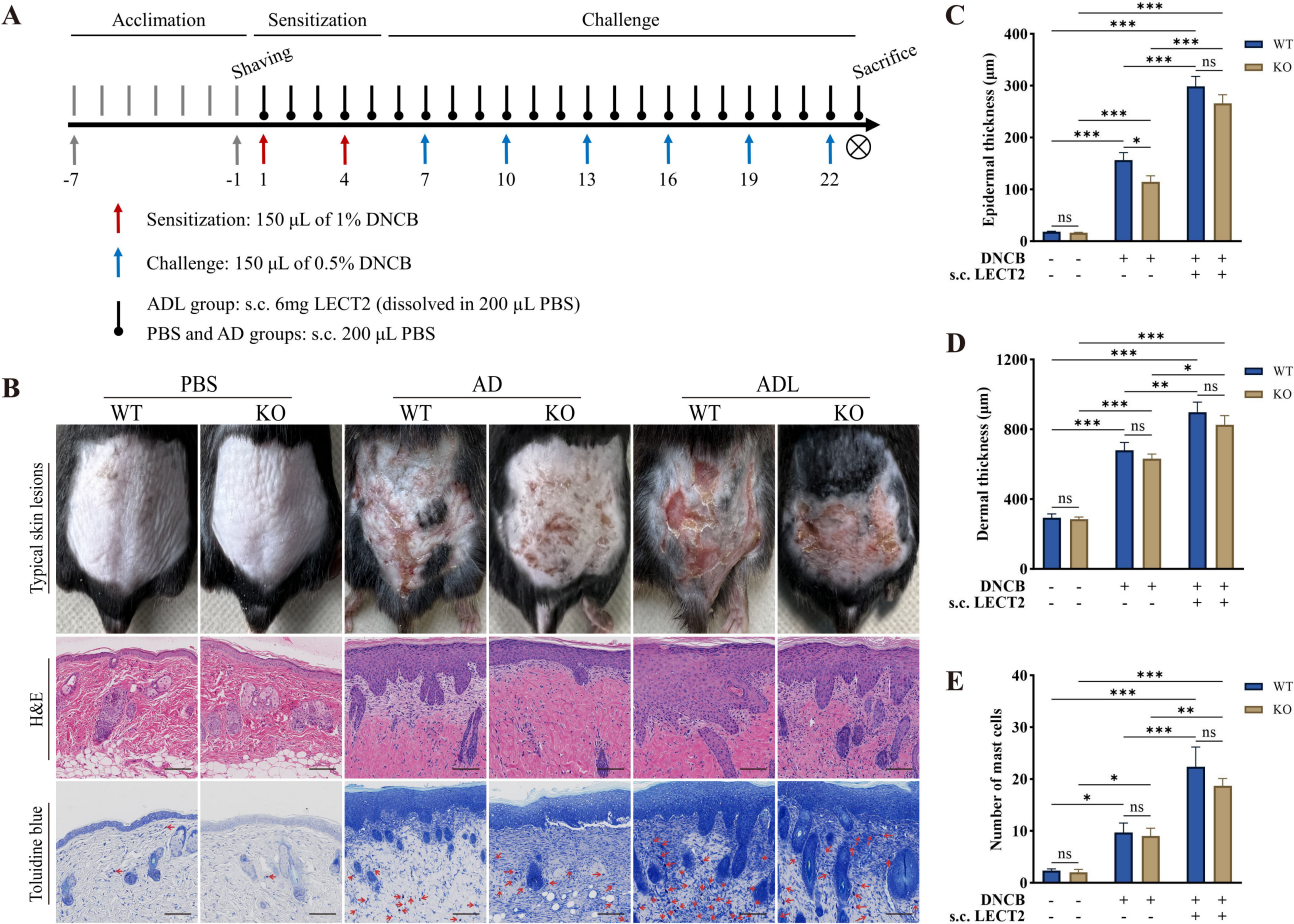
Effect of LECT2 on skin manifestations and pathological changes in DNCB-induced AD-like skin lesions. **(A)** Scheme of the animal experimental procedure. **(B)** Typical skin lesions on day 23 of the animal experiment and histological analysis with hematoxylin and eosin (H&E) staining showing epidermal and dermal structures and toluidine blue staining highlighting mast cell infiltration. **(C)** Determination of epidermal thickness. **(D)** Determination of dermal thickness. **(E)** Number of mast cells in slides under 200× magnification field of view, with toluidine blue-stained mast cells marked with red arrows, and the number of mast cells expressed as the mean total count in three fields of view. The data shown represent mean ± SEM and are representative of at least three independent experiments. Scale bar: 100 μm. *P < 0.05, **P < 0.01, ***P < 0.001; ns, not significant.

DNCB is a semi-antigen that, when bound to skin tissue proteins, can become a full antigen triggering an immune response (33). Repeated application of DNCB could lead to a shift from a T-helper (Th) 1 cell-mediated delayed hypersensitivity response to a Th2 cell-mediated chronic inflammatory response, thus emulating human AD (34). In this study, on day 23 of the animal experiment, DNCB-induced mice showed obvious AD physical signs, such as erythema, thickening, dryness, and lichenification. The ADL group mice exhibited more severe AD physical signs due to s.c. LECT2 treatment. In contrast, the absence of the LECT2 gene in KO mice reduced the severity of these symptoms. Typical skin lesions on day 23 of the animal experiment are shown in **Figure 1B**.

### 2.2 LECT2 exacerbates pathological changes in DNCB-induced AD-like skin lesions

AD-like skin lesions are characterized by thickening of the epidermis and dermis (35). We analyzed the effect of LECT2 on these thickness changes in DNCB-induced AD-like skin lesions using H&E staining. Compared to the AD group, s.c. LECT2 significantly increased the thickness of both the epidermis and dermis in the skin lesions. In the AD group, the absence of the LECT2 gene resulted in a relative decrease in epidermal thickness (**Figures 1C, D**). Mast cell infiltration is a pathophysiologic feature of several allergic diseases, including AD (36). Mast cells possess the FcϵRI receptor (a high-affinity IgE receptor), and their combination can lead to the degranulation and the release of a large number of inflammatory mediators (37). We analyzed the number of mast cells in DNCB-induced AD-like skin lesions by toluidine blue staining. The number of mast cells increased in the skin lesions and was further elevated after s.c. LECT2 treatment (**Figure 1E**).

### 2.3 LECT2 decreases barrier protein expression and increases inflammatory factor expression in DNCB-induced AD-like skin lesions

**Figure 2:**
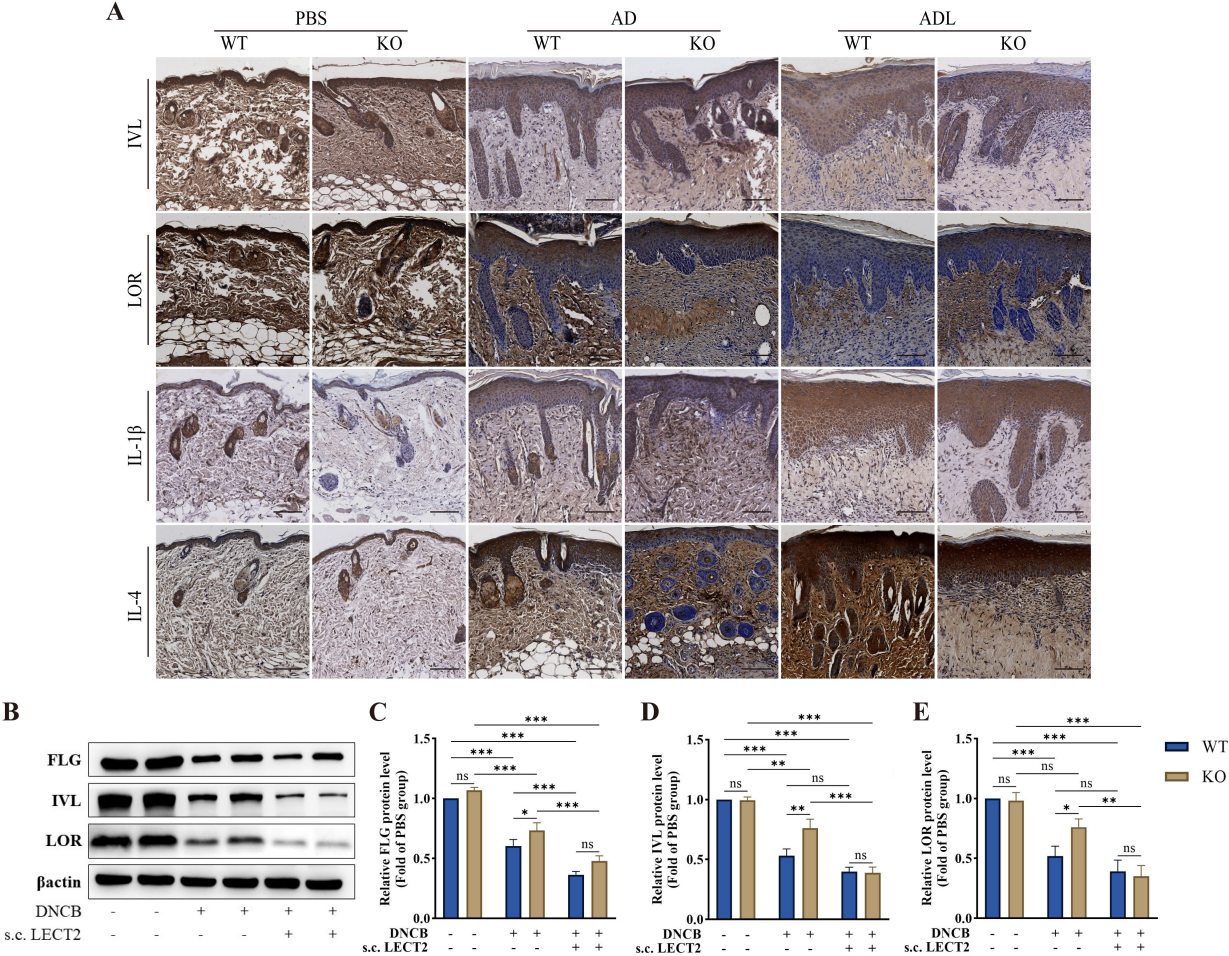
Effect of LECT2 on the expression of the barrier proteins (FLG, IVL, and LOR) and the inflammatory factors (IL-1β and IL-4) in DNCB-induced AD-like skin lesions. **(A)** The immunostaining for each protein was brown, with darker colors representing higher expression of the protein. **(B)** Western Blot analysis of the levels of barrier proteins **(C)** FLG, **(D)** IVL, and **(E)** LOR in DNCB-induced AD-like skin lesions. WT indicates wild-type mice, and KO indicates knockout mice. The data shown represent mean ± SEM and are representative of at least three independent experiments. Scale bar: 100 μm. *P < 0.05, **P < 0.01, ***P < 0.001; ns, not significant.

Barrier proteins, including FLG, IVL, and LOR are crucial structural components of the skin. Reduced expression of these proteins leads to skin barrier dysfunction, resulting in dry skin, allergen leakage, and skin microecological imbalance, further stimulating the immune system and causing immune dysfunction (6–8). We analyzed the effect of LECT2 on the expression of barrier proteins and inflammatory factors in DNCB-induced AD-like skin lesions using immunohistochemistry and Western Blot. The expression of barrier proteins FLG, IVL, and LOR decreased, while the expression of inflammatory factors IL-1β and IL-4 increased in the skin lesions, after s.c. LECT2 treatment, the expression of barrier proteins FLG, IVL, and LOR was further reduced, and the expression of inflammatory factors IL-1β and IL-4 was further elevated. In contrast, in the AD group, the absence of the LECT2 gene was associated with relatively high expression of barrier proteins FLG, IVL, and LOR and relatively low expression of the inflammatory factors IL-1β and IL-4. The immunohistochemical images and Western Blot analysis of each protein in DNCB-induced AD-like skin lesions are shown in **Figures 2A–E**.

### 2.4 LECT2 increases inflammatory factor levels in DNCB-induced AD-like skin lesions and DNCB-induced mice serum

**Figure 3:**
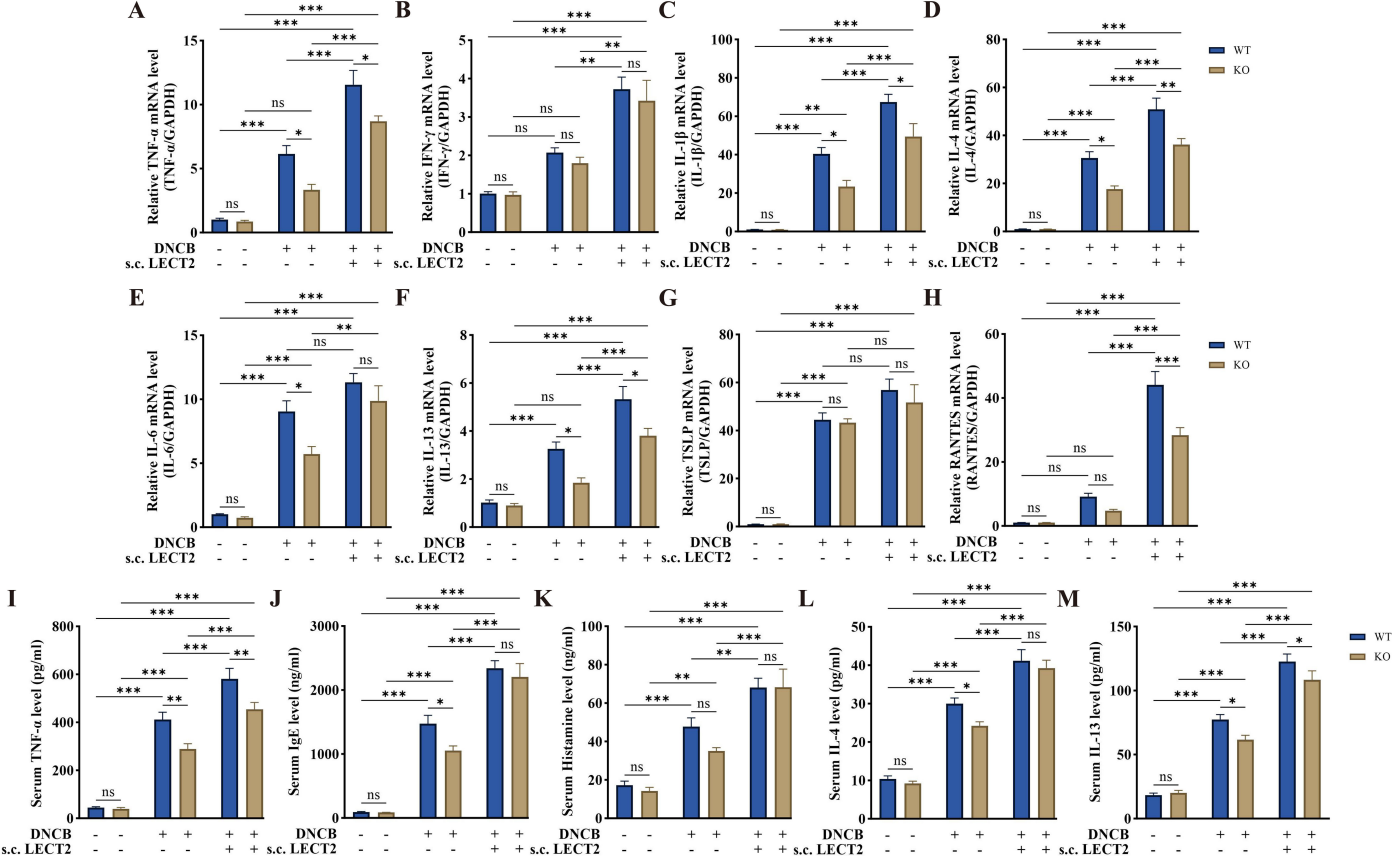
Effect of LECT2 on inflammatory factor levels in DNCB-induced AD-like skin lesions and DNCB-induced mice serum. The mRNA levels of **(A)** TNF-α, **(B)** IFN-γ, **(C)** IL-1β, **(D)** IL-4, **(E)** IL-6, **(F)** IL-13, **(G)** TSLP, and **(H)** RANTES were analyzed in DNCB-induced AD-like skin lesions by RT-qPCR. The protein levels of **(I)** TNF-α, **(J)** IgE, **(K)** histamine, **(L)** IL-4, and **(M)** IL-13 were analyzed in DNCB-induced mouse serum by ELISA. The data shown represent mean ± SEM and are representative of at least three independent experiments. *P < 0.05, **P < 0.01, ***P < 0.001; ns, not significant.

The dysfunction of the immune system in AD is associated with Th2 cell-mediated immune responses (38). Cytokines such as IL-4 secreted by Th2 cells can promote B cells to secrete IgE, which binds to mast cell surface receptors FcϵRI, stimulating mast cell degranulation and releasing inflammatory mediators such as histamine (39–41). TSLP, an important pathogenic molecule, and therapeutic target in AD, is mainly secreted by keratinocytes. It acts as a regulator of immune response and can induce Th2 cell-mediated immune responses (42, 43). We analyzed the mRNA levels of TNF-α, IFN-γ, IL-1β, IL-4, IL-6, IL-13, TSLP, and RANTES in DNCB-induced AD-like skin lesions using RT-qPCR (**Figures 3A–H**), and the protein levels of TNF-α, IgE, histamine, IL-4, and IL-13 in DNCB-induced mouse serum using ELISA (**Figures 3I–M**). The mRNA levels of TNF-α, IL-1β, IL-4, IL-6, IL-13, and TSLP were increased in the skin lesions, with the levels of all inflammatory factors except TSLP further increased after s.c. LECT2 treatment. Similarly, the protein levels of TNF-α, IgE, histamine, IL-4, and IL-13 were increased in serum and were further elevated after s.c. LECT2 treatment. In contrast, in the AD group, the absence of the LECT2 gene resulted in relatively decreased mRNA levels of TNF-α, IL-1β, IL-4, IL-6, and IL-13 in DNCB-induced AD-like skin lesions, and relatively reduced protein levels of TNF-α, IgE, IL-4, and IL-13 in DNCB-induced mice serum.

### 2.5 LECT2 increases inflammatory factor levels in TNF-α/IFN-γ-induced HaCaT cells

**Figure 4:**
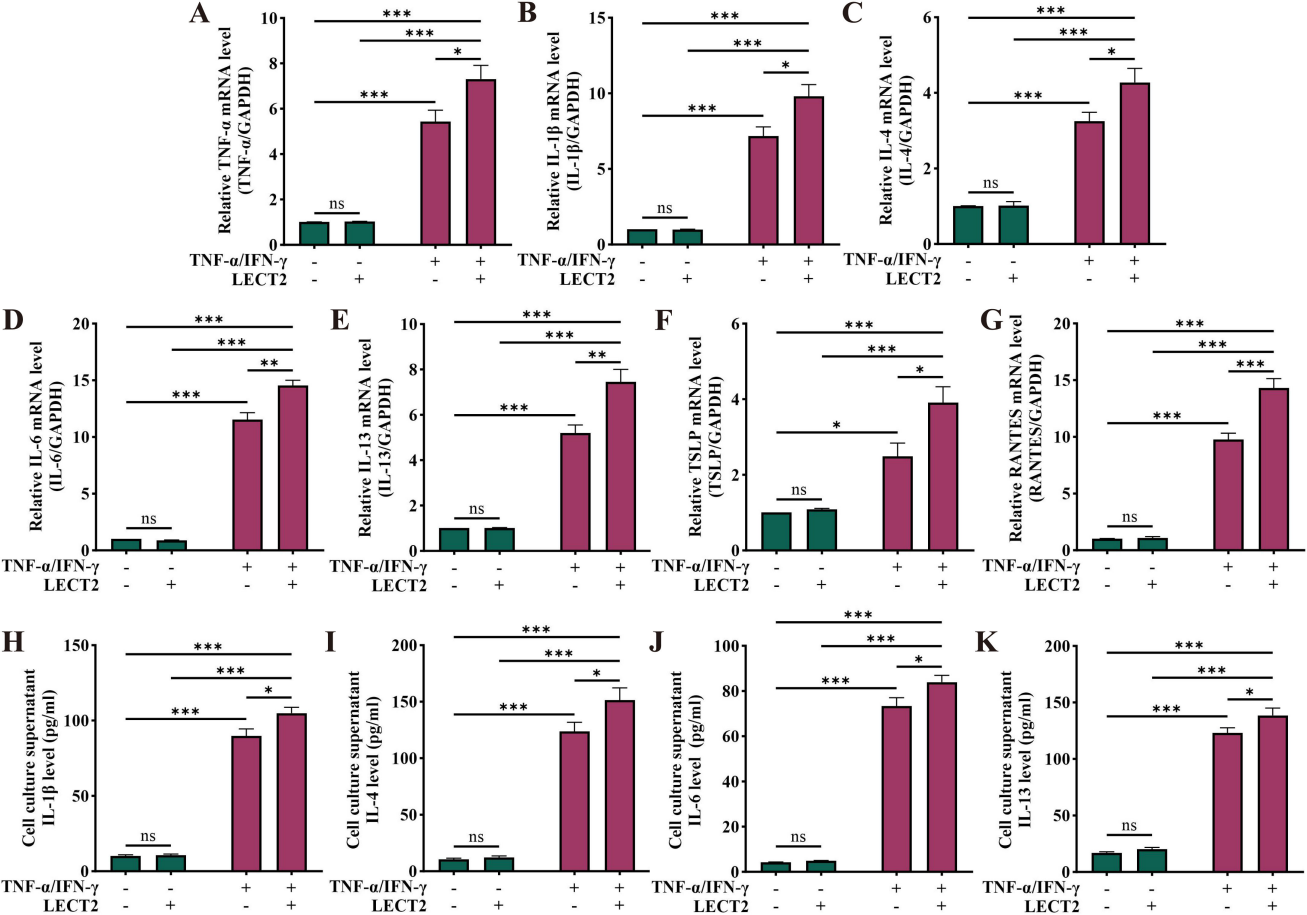
Effect of LECT2 on inflammatory factor levels in TNF-α/IFN-γ-induced HaCaT cells. The mRNA levels of **(A)** TNF-α, **(B)** IL-1β, **(C)** IL-4, **(D)** IL-6, **(E)** IL-13, **(F)** TSLP, and **(G)** RANTES were analyzed in TNF-α/IFN-γ-induced HaCaT cells by RT-qPCR. The protein levels of **(H)** IL-1β, **(I)** IL-4, **(J)** IL-6, and **(K)** IL-13 were analyzed in TNF-α/IFN-γ-induced HaCaT cells culture supernatant by ELISA. HaCaT cells were treated with LECT2 (400 ng/ml) for 1 hour to extract total RNA for RT-qPCR, and the cells were treated for 24 hours to collect cell culture supernatant for ELISA. The data shown represent mean ± SEM and represent at least three independent experiments. *P < 0.05, **P < 0.01, ***P < 0.001; ns, not significant.

To further examine the effect of LECT2 on AD, we constructed an AD cell model by inducing HaCaT cells with TNF-α/IFN-γ. We analyzed the mRNA levels of TNF-α, IL-1β, IL-4, IL-6, IL-13, TSLP, and RANTES in TNF-α/IFN-γ-induced HaCaT cells by RT-qPCR (**Figures 4A–G**), and the protein levels of IL-1β, IL-4, IL-6, and IL-13 in the cell culture supernatant of TNF-α/IFN-γ-induced HaCaT cells by ELISA (**Figures 4H–K**). The mRNA levels of TNF-α, IL-1β, IL-4, IL-6, IL-13, TSLP, and RANTES were increased in the AD cell model constructed by TNF-α/IFN-γ-induced HaCaT cells, and protein levels of IL-1β, IL-4, IL-6, and IL-13 were also elevated. After treatment of the AD cell model with LECT2, the levels of the above inflammatory factors were further increased. However, treatment of healthy HaCaT cells with LECT2 did not significantly alter the levels of these inflammatory factors.

### 2.6 LECT2 decreases barrier protein levels in TNF-α/IFN-γ-induced HaCaT cells

**Figure 5:**
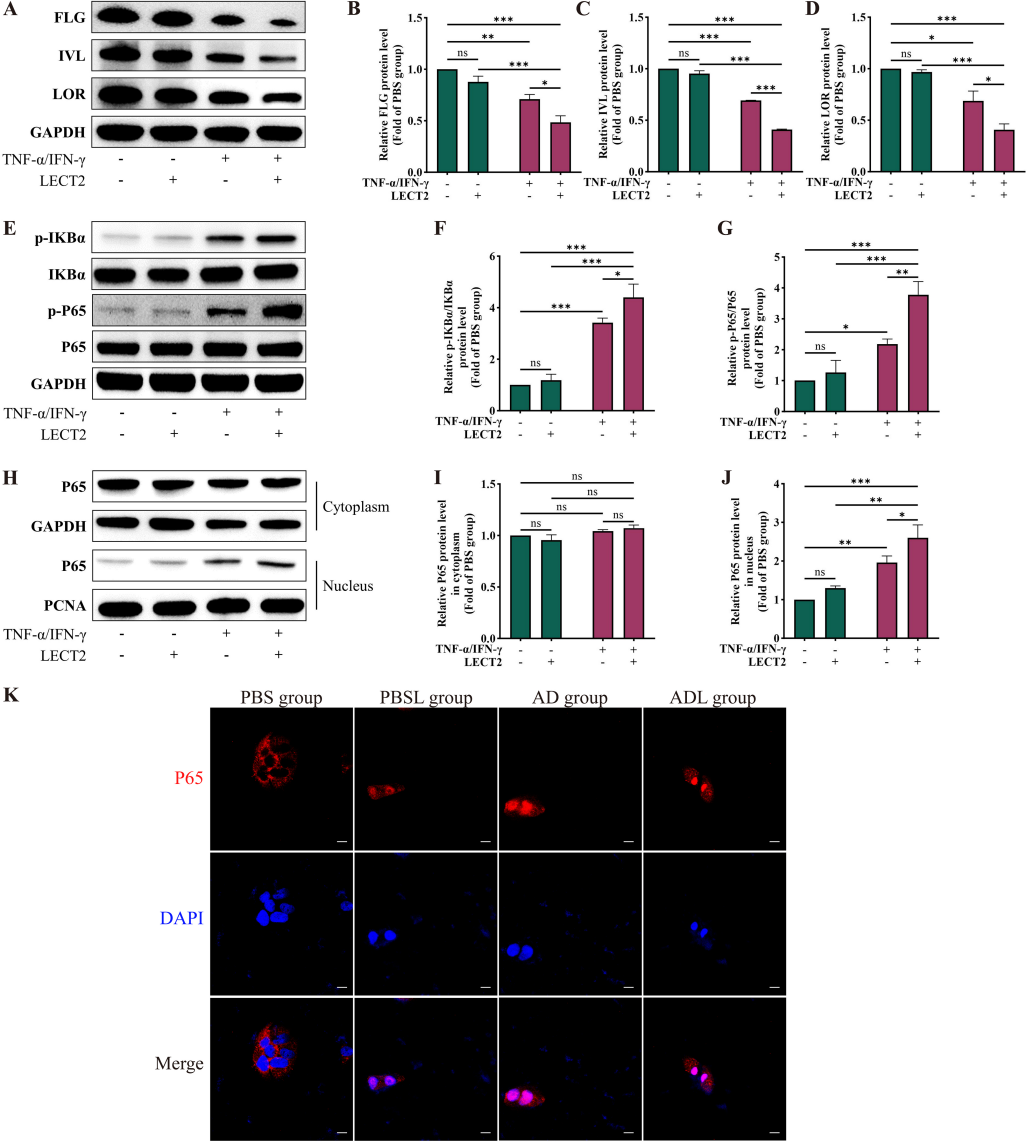
Effect of LECT2 on barrier protein levels and NF-κB signaling pathway in TNF-α/IFN-γ-induced HaCaT cells. **(A)** Western Blot analysis of the levels of barrier proteins **(B)** FLG, **(C)** IVL, and **(D)** LOR in TNF-α/IFN-γ-induced HaCaT cells. **(E)** Western Blot analysis of the protein levels of **(F)** p-IKBα/IKBα, and **(G)** p-P65/P65 in total proteins of TNF-α/IFN-γ-induced HaCaT cells. **(H)** Western Blot analysis of the protein levels of **(I)** P65 in cytoplasmic proteins and **(J)** P65 in nuclear proteins of TNF-α/IFN-γ-induced HaCaT cells. **(K)** Immunofluorescence staining for P65 protein (labeled with Cy3, red). HaCaT cells were counterstained with DAPI (blue). HaCaT cells were treated with LECT2 (400 ng/ml) for 24 hours to extract total, cytoplasmic and nuclear proteins for Western Blot. The data shown represent mean ± SEM and represent at least three independent experiments. Scale bar: 20 μm *P < 0.05, **P < 0.01, ***P < 0.001; ns, not significant.

Th2 cytokines, such as IL-4 and IL-13, can downregulate the levels of barrier proteins FLG, IVL, and LOR in AD (14–17). We analyzed the levels of these barrier proteins in TNF-α/IFN-γ-induced HaCaT cells by Western Blot (**Figures 5A–D**). The levels of barrier proteins FLG, IVL, and LOR were decreased in the AD cell model constructed from TNF-α/IFN-γ-induced HaCaT cells. After treatment of the AD cell model with LECT2, the FLG, IVL, and LOR levels were further decreased. However, treatment of healthy HaCaT cells with LECT2 showed no statistically significant difference in the levels of these barrier proteins, although they were reduced.

### 2.7 LECT2 further activates the NF-κB signaling pathway in TNF-α/IFN-γ-induced HaCaT cells

Activation of the NF-κB signaling pathway plays an essential role in various acute and chronic inflammatory and allergic diseases, including AD (44–47). In adipocytes, LECT2 can activate the NF-κB signaling pathway and promote the expression of inflammatory factors TNF-α and IL-6. In macrophage, LECT2 can activate the NF-κB signaling pathway and enhance their phagocytic activity (26, 48). Therefore, we first analyzed the phosphorylation levels of Inhibitors of NF-κB (IKB)α and P65 proteins in TNF-α/IFN-γ-induced HaCaT cells by Western Blot (**Figures 5E–G**). The phosphorylation levels of IKBα and P65 proteins were increased in the total proteins of the AD cell model constructed from TNF-α/IFN-γ-induced HaCaT cells. After treatment of the AD cell model with LECT2, the phosphorylation levels of IKBα and P65 proteins were further increased. The P65 protein needs to be translocated to the nucleus to function after dissociation from the IKB protein in the cytoplasm (49). Therefore, we extracted cytoplasmic and nuclear proteins from TNF-α/IFN-γ-induced HaCaT cells and analyzed the protein level of P65 by Western Blot (**Figures 5H–J**). In the cytoplasm, there was no statistically significant difference in the protein level of P65 before and after treatment with LECT2 in the AD cell model constructed from TNF-α/IFN-γ-induced HaCaT cells. However, in the nucleus, the protein level of P65 was increased in the AD cell model and further increased after treatment of the AD cell model with LECT2. Nevertheless, treatment of healthy HaCaT cells with LECT2 showed no statistically significant differences in the phosphorylation levels of IKBα and P65 proteins in total protein, nor the protein level of P65 in cytoplasmic and nuclear proteins.

We also performed immunofluorescence staining of P65 protein to observe the nuclear translocation of P65 protein (**Figure 5K**). In healthy HaCaT cells, most of the P65 protein was located in the cytoplasm. In the AD cell model, the translocation of P65 protein from the cytoplasm to the nucleus was increased, and the nuclear translocation of P65 protein was further increased after treatment of the AD cell model with LECT2.

## 3 Discussion

Our study demonstrates that LECT2 exacerbates AD by impairing skin barrier function and increasing inflammatory responses through the activation of the NF-κB signaling pathway. This finding provides new insights into the molecular mechanisms underlying AD and suggests potential therapeutic targets for this chronic inflammatory skin disease.

AD is characterized by impaired skin barrier function and immune system dysfunction (6–9). The skin barrier impairment is marked by decreased expression of the barrier proteins FLG, IVL, and LOR (6, 7). Our study found that the expression of FLG, IVL, and LOR was decreased in the AD mouse model (DNCB-induced KO and WT mice) via immunohistochemistry and Western Blot analysis, which was further decreased by LECT2 treatment. Similarly, decreased FLG, IVL, and LOR expression were observed in the AD cell model (TNF-α/IFN-γ-induced HaCaT cells), as shown by Western Blot, with LECT2 treatment exacerbating these reductions.

Our animal studies focused on IVL and LOR as representative barrier proteins due to their well-documented roles in skin barrier function and robust expression patterns in murine models of atopic dermatitis (16). While FLG is also a crucial barrier protein, its expression can be highly variable and influenced by different factors in murine models (50), making it less consistent with our study’s specific aims. Therefore, we concentrated on IVL and LOR to provide clear and consistent data on barrier protein expression. We acknowledge the importance of FLG and will consider its inclusion in future studies for a more comprehensive analysis.

Immune system dysfunction in AD manifests as a predominance of Th2 cell-mediated immune responses (9, 10). Th2 cells produce inflammatory factors such as IL-4, IL-6, and IL-13, and induce IgE class-switching, mediating mast cell degranulation and the release of histamine, cytokines, and chemokines (51). IL-4 promotes B cell differentiation and IgE production, leading to allergen-specific responses that exacerbate AD. This process is well-documented and clarifies the connection between IL-4 and the exacerbation of AD symptoms (52, 53). IL-4 induces Th0 to Th2 differentiation, and together with IL-13 downregulates the expression of barrier proteins in the skin (54, 55). TSLP, an epithelium-derived pro-inflammatory cytokine, plays a crucial role in AD deterioration by interacting with a wide range of immune cells (42, 56). Our study found increased mast cell infiltration and elevated levels of inflammatory factors (TNF-α, IL-1β, IL-4, IL-6, IL-13, TSLP, IgE, and histamine) in the AD mouse model. LECT2 treatment further increased these factors while KO mice showed relatively low levels. Similar increases in inflammatory factors were observed in the AD cell model treated with LECT2.

We measured mRNA levels of proinflammatory molecules in the skin to reflect local tissue-specific expression changes directly at the site of inflammation. This approach allowed us to capture gene expression alterations in response to LECT2 treatment. Conversely, protein levels were measured in the serum to evaluate the systemic inflammatory response, providing insight into the overall inflammatory status of the organism. Future studies will include protein measurements of proinflammatory molecules in the skin to gain a more comprehensive understanding of the local inflammatory response and its interplay with systemic inflammation.

The differential expression of TSLP between the mouse model and HaCaT cells likely reflects species-specific responses and localized cellular environments. This suggests that different models can show varying levels of TSLP due to their unique biological contexts. Therefore, the complexity of AD pathogenesis requires the use of multiple models to capture these nuances. This added layer of complexity is necessary to provide a comprehensive understanding of AD and its underlying mechanisms.

The unchanged TSLP levels in LECT2 knockout mice suggest that TSLP is not directly regulated by LECT2 in this model. This raises the intriguing question of whether TSLP is a cause or an effect of atopic dermatitis. In the LECT2 knockout mouse model, the DNCB-induced expression of TSLP was unaffected, yet there was a reduction in lesion severity. This discrepancy suggests that TSLP may act independently of LECT2 in certain pathways or stages of AD pathogenesis. Therefore, the role of TSLP in AD might be more complex and multifaceted, potentially acting both as a mediator and a consequence of inflammatory processes depending on the context (57).

LECT2, first identified as a chemokine for neutrophil migration (58, 59) is associated with immune diseases like non-alcoholic steatohepatitis (NASH), atherosclerosis (AS) (27) and cholestatic liver disease. High LECT2 levels correlate with disease severity, suggesting its role as a potential biomarker and therapeutic target (29, 60–62). Our study aligns with previous findings, showing that LECT2 exacerbates AD-like responses, and highlights the potential of targeting LECT2 for AD management.

The NF-κB signaling pathway is a classical inflammatory response pathway, upregulated in chronic inflammatory diseases (63). Meanwhile, several pharmacological studies have also demonstrated that inhibition of NF-κB signaling pathway activation can play a therapeutic role in AD-like models *in vivo* and *in vitro* (17, 64–66). LECT2 is also closely associated with the activation of the NF-κB pathway. Shen et al. found that LECT2 upregulated the DNA-binding activity and nuclear translocation of NF-κB P65 protein and enhanced the killing of H. pylori by macrophages (26). Jung et al. reported that LECT2 enhances the phosphorylation of IKB and NF-κB P65 proteins, promotes TNF-α and IL-6 expression, and stimulates inflammation in adipocytes (48). Therefore, we hypothesized that LECT2 exacerbates the responses in an AD-like model by activating the NF-κB signaling pathway. In the present study, we found increased phosphorylation levels of IKB and NF-κB P65 proteins in the AD cell model treated with LECT2. This activation, confirmed by Western Blot and immunofluorescence, likely mediates the observed decreases in barrier protein expression and increases in inflammatory cytokine production. NF-κB P65 protein translocates to the nucleus after dissociation from IKB in the cytoplasm, where it binds to specific DNA sequences and activates gene transcription, leading to the production of inflammatory mediators (49). Our findings indicate that LECT2 enhances NF-κB activation in the presence of TNF-α/IFN-γ, suggesting a synergistic effect. LECT2 may not independently activate NF-κB in HaCaT cells but rather amplifies existing inflammatory signals. This suggests that *in vivo* activation of keratinocytes by TNF-α or IFN-γ could enhance the expression and effects of LECT2.

However, since LECT2 is primarily produced by the liver, its expression is regulated by systemic inflammatory signals. Inflammatory cytokines such as TNF-α and IFN-γ can stimulate the liver to produce LECT2 by binding to their receptors on hepatocytes and activating intracellular signaling pathways, including NF-κB, which upregulate the transcription of the LECT2 gene. Understanding this systemic regulation is crucial for developing therapeutic strategies targeting LECT2 in AD.

Interestingly, we found no statistically significant differences in the expression of inflammatory factors and barrier proteins after LECT2 treatment of healthy HaCaT cells. This may be due to the chemotactic properties of LECT2, which is ineffective in recruiting inflammatory factors without an existing inflammatory response. LECT2, as a pleiotropic protein, may exacerbate the responses in AD-like models potentially through other mechanisms. Several studies have shown that LECT2 receptors on macrophages promote phenotypic switching, participating in the inflammatory response in a variety of immune diseases, such as bacterial infections, NASH, AS, ovarian cancer, and cholestatic liver disease (24, 31, 60, 67, 68). This phenotypic transformation of macrophages is also present in a variety of atopic diseases, including AD (69, 70). Therefore, this is the direction of our team’s research in the subsequent work.

While our study provides valuable insights, it has limitations. Although DNCB is widely used to induce *in vivo* models of AD, transcriptome analysis suggests that the model has approximately 40% homology to human AD (71), which may limit the generalization of the conclusions to humans. In addition, investigating the effects of LECT2 alone was beyond the initial scope, which focused on combined effects with DNCB-induced AD. However, our *in vitro* results suggest that LECT2 enhances inflammation within an existing inflammatory context, but overexpression of LECT2 alone did not induce AD-like symptoms. This indicates that LECT2 exacerbates but does not independently initiate AD, aligning with our hypothesis that LECT2 enhances existing inflammatory conditions. Future studies should explore the direct effects of LECT2 *in vivo* to fully understand its role in AD pathogenesis and clarify whether LECT2 alone can induce inflammatory responses or if its exacerbating effects depend on pre-existing inflammation. Furthermore, we did not measure serum LECT2 levels in KO mice, focusing instead on the absence of LECT2 and its resultant effects. Future research should address this gap and explore agents that can effectively reduce LECT2 levels.

In conclusion, our study elucidates the role of LECT2 in exacerbating AD by impairing skin barrier function and promoting inflammation through the NF-κB signaling pathway. These findings suggest that targeting LECT2 or its signaling pathways could offer new therapeutic strategies for managing AD. Future research should explore the detailed mechanisms of LECT2-mediated macrophage activation and its broader implications in AD and other inflammatory diseases.

## 4 Materials and methods

### 4.1 Animal experiment

Wild-type (WT) mice were purchased from Vital River Laboratory Animal Technology Co., Ltd. (Zhejiang, China). Knockout (KO) mice were donated by Professor Jiong Chen, School of Marine Sciences, Ningbo University. All mice had a C57BL/6 genetic background and were maintained under specific pathogen-free (SPF) conditions. WT and KO mice were housed under an SPF-grade barrier in the Laboratory Animal Center of Ningbo University, with a controlled environment of a constant light cycle (lights on at 8:00 am and off at 8:00 pm), temperature (20-24°C), and humidity (40-70%). The mice had ample space to move around and free access to sterile water and food. All experimental procedures were conducted according to the Guidelines for the Care and Use of Laboratory Animals issued by the National Institutes of Health and were reviewed and approved by the Ethics Committee of Ningbo University Laboratory Animal (NBU20220287).

The AD mouse model was established based on previous study methods (72–74). Briefly, DNCB (138630, Sigma-Aldrich, Steinheim, Germany) powder was solubilized in a solvent mixture of acetone and olive oil (3:1; V/V). After the mice were acclimatized within the SPF-grade barrier for one week, the backs of the mice were depilated using a razor and depilatory cream. The next day, the sensitization process began by applying 150 µL of 1% DNCB (W/V) to the dorsal skin of the mice every three days, for a total of two applications. This was followed by the challenge process, applying 150 µL of 0.5% DNCB (W/V) to the dorsal skin of the mice every three days, for a total of six applications.

Recombinant mouse LECT2 was purchased from CUSABIO Bioengineering Co., Ltd. (CSB-YP012855MO; Wuhan, China) and produced using a yeast culture system. LECT2 was dissolved in PBS and stored at 4°C for up to three days. Beginning on the first day of sensitization and continuing until the day the mice were sacrificed, the dorsal skin of the mice received a subcutaneous injection (s.c.) of 6 mg LECT2 (dissolved in 200 µL PBS) every afternoon or 30 minutes after application of DNCB. The mice’s serum and dorsal skin were collected 30 minutes after the last injection for subsequent experiments.

### 4.2 Grouping of animal experiment

Male mice (6-8 weeks old; 22 ± 2 g) were randomly divided into three groups, each containing six WT mice and six KO mice): (1) PBS group, where the dorsal skin was applied with an equal volume of solvent and received subcutaneous PBS (s.c. PBS); (2) AD group, where the dorsal skin was DNCB-sensitized and challenged and received subcutaneous PBS (s.c. PBS); and (3) ADL group, where the dorsal skin was DNCB-sensitized and challenged and received subcutaneous LECT2 (s.c. LECT2). The scheme of the animal experimental procedure is shown in **Figure 1A**.

### 4.3 Cell culture

HaCaT cells were purchased from Pricella Biotechnology Co., Ltd. (CL-0090; Wuhan, China) and cultured in high glucose Dulbecco’s modified Eagle’s medium (DMEM) supplemented with 10% fetal bovine serum (FBS; V/V) and 1% penicillin-streptomycin antibiotic (V/V) at 37°C in a humidified incubator with 5% CO_2_. The AD cell model was established based on previous study methods (73, 75). Briefly, HaCaT cells were seeded into six-well plates, and when the cells reached 60-70% confluence, they were treated with TNF-α (10 ng/mL; 021825, PeproTech, Rocky Hill, NJ, USA) and IFN-γ (10 ng/mL; 091927, PeproTech, Rocky Hill, NJ, USA) for 24 hours to establish the AD cell model. The medium was then replaced with serum-free DMEM with the addition of LECT2 (400 ng/mL) or an equal volume of PBS. Continue to treat the cells for 1 hour to extract total RNA for RT-qPCR, and for 24 hours to extract proteins for Western Blot or collect cell culture supernatant for ELISA.

Recombinant human LECT2 was purchased from R&D Systems, Inc. (9908-LC; Minneapolis, MN, USA) and produced using a mammalian culture system with the HEK293 cell line.

### 4.4 Grouping of cell experiment

HaCaT cells were divided into four groups: (1) PBS group (without TNF-α/IFN-γ; treated with PBS); (2) PBSL group (without TNF-α/IFN-γ; treated with LECT2); (3) AD group (treated with TNF-α/IFN-γ; treated with PBS); (4) ADL group (treated with TNF-α/IFN-γ; treated with LECT2).

### 4.5 Histological analysis and immunohistochemistry

Mouse dorsal skin tissue specimens were fixed in 4% paraformaldehyde (P1110, Solarbio, Beijing, China) for 24 hours, then dehydrated in graded ethanol, cleared in xylene, and finally embedded in paraffin. Using the frozen sectioning machine (CM1860, Leica, Wetzlar, Germany), 4 µm slides were made perpendicular to the longitudinal axis of the tissue specimen. The slides were sequentially submerged in xylene, graded ethanol, and distilled water for rehydration. Hematoxylin and eosin (H&E; G1100, Solarbio, Beijing, China) staining was used for epidermal and dermal thickness analysis, and toluidine blue (G3670, Solarbio, Beijing, China) staining was used for mast cell infiltration analysis. Digital images of the slides were captured at 200× magnification using the microscope (DM IL LED, Leica, Wetzlar, Germany). The thickness of the epidermis and dermis and the number of mast cells were obtained by averaging the measurements of each mouse in three digital images using iSolution (Version 8.1, IMT i-Solution Inc., Burnaby, BC, Canada).

To assess the expression of inflammatory factors and barrier proteins in the dorsal skin tissue specimens, the slides were rehydrated, antigenically repaired, inactivated with endogenous peroxidase, blocked, and then incubated with primary antibodies: Involucrin (1:50; sc-21748, Santa Cruz Biotechnology, Santa Cruz, CA, USA), Loricrin (1:200; 55439-1-AP, ProteinTech, Wuhan, China), IL-1β (1:100; AF5103, Affinity biosciences, Changzhou, China), or IL-4 (1:100; AF5142, Affinity biosciences, Changzhou, China) primary antibody overnight at 4°C. This was followed by incubation with HRP-conjugated secondary antibody (ProteinTech, Wuhan, China) for 1 hour at room temperature, and after Diaminobenzidine (DA1010, Solarbio, Beijing, China) staining and hematoxylin (G1100, Solarbio, Beijing, China) restaining, digital images were captured under the microscope (DM IL LED, Leica, Wetzlar, Germany).

### 4.6 Real-time quantitative reverse transcription polymerase chain reaction

All Kits were used following the manufacturer’s protocol. Total RNA was extracted from mouse dorsal skin tissue specimens or HaCaT cells using the Universal RNA Extraction Kit (9767, TaKaRa, Shiga, Japan). Total RNA was quantified using the NanoDrop One (Thermo Fisher Scientific, Wilmington, DE, USA), and cDNA was obtained by reverse transcribing 1000 ng of RNA in a 20 μL system using the RT Master Mix (RR036A, TaKaRa, Shiga, Japan). Quantitative real-time PCR analysis of gene expression was performed using the Tli RNaseH Plus (RR820A, TaKaRa, Shiga, Japan) in the LightCycler 480 (Roche Diagnostics, Basel, Switzerland). The qPCR products were further confirmed by melting curve analysis. The levels of target genes were normalized relative to the GAPDH gene level, and the relative RNA level fold was calculated by the 2^-ΔΔCT^ method. The primer sequences are shown in **Supplementary Tables S1** and **S2**.

### 4.7 Enzyme-linked immunosorbent assay

The levels of IgE (E-EL-M3034, Elabscience, Wuhan, China), Histamine (E-EL-0032c, Elabscience, Wuhan, China), TNF-α (KE10002, ProteinTech, Wuhan, China), IL-4 (KE10010, ProteinTech, Wuhan, China), and IL-13 (KE10021, ProteinTech, Wuhan, China) in mouse serum, as well as the levels of IL-1β (KE00021, ProteinTech, Wuhan, China), IL-4 (KE00016, ProteinTech, Wuhan, China), IL-6 (E-EL-H6156, Elabscience, Wuhan, China), and IL-13 (E-EL-H0104, Elabscience, Wuhan, China) in HaCaT cell culture supernatant, were measured using commercial ELISA kits according to the manufacturer’s protocol.

### 4.8 Western blot analysis

Total proteins from mouse dorsal skin tissue specimens and HaCaT cells were extracted using RIPA lysis buffer (P0013B, Beyotime, Shanghai, China). Cytoplasmic and nuclear proteins from HaCaT cells were extracted using the Cytoplasmic and Nuclear Proteins Extraction Kit (P0027, Beyotime, Shanghai, China). All protein extraction procedures were supplemented with protease and phosphatase inhibitors (P1045, Beyotime, Shanghai, China) and were carried out at low temperatures throughout.

Protein specimens (20-30 μg) were separated using the TGX Stain-Free™ FastCast™ Acrylamide Kit (1610183, Bio-Rad, Hercules, CA, USA) in the Mini-PROTEAN Tetra Handcast Systems (Bio-Rad, Hercules, CA, USA) and transferred to PVDF membrane (pore size 0.22/0.45 μm, Millipore, Billerica, MA, USA). The PVDF membrane was blocked using the QuickBlock™ Western Blocking Solution (P0252, Beyotime, Shanghai, China) for 15 minutes. After washing, the membrane was incubated overnight at 4°C on a shaker with the following primary antibodies: GAPDH (1:5000; 60004-1-Ig, ProteinTech, Wuhan, China), PCNA (1:6000; 60097-1-Ig, ProteinTech, Wuhan, China), Filaggrin (1:1000; GTX23137 GeneTex, Shenzhen, China), Involucrin (1:2000; sc-21748, Santa Cruz Biotechnology, Santa Cruz, CA, USA), Loricrin (1:1000; 55439-1-AP, ProteinTech, Wuhan, China), NF-κB P65 (1:2000; ab32536, Abcam, Cambridge, MA, USA), NF-κB p-P65 (1:1000; ab76302, Abcam, Cambridge, MA, USA), IKBα (1:1000; ab32518, Abcam, Cambridge, MA, USA), or p-IKBα (1:1000; ab133462, Abcam, Cambridge, MA, USA).

The primary antibody was then recovered, and the PVDF membranes were washed and then incubated with the HRP-conjugated secondary antibodies (ProteinTech, Wuhan, China) for 1 hour at room temperature on a shaker. The membranes were washed again, and the Clarity Western ECL Substrate (1705061, Bio-Rad, Hercules, CA, USA) was evenly applied dropwise. The PVDF membranes were visualized using the ChemiDoc MP Imaging System (Bio-Rad, Hercules, CA, USA). Optical density analysis was performed using Image J (Version 1.53c, National Institutes of Health, Bethesda, MD, USA) with GAPDH or PCNA as internal reference proteins.

### 4.9 Immunofluorescence staining

HaCaT cells were seeded at a density of 5×10^4^ per dish in laser confocal Petri dishes (YA0572, Solarbio, Beijing, China), and treated according to their respective groupings. The treated cells were washed with PBST and fixed with 4% paraformaldehyde (P1110, Solarbio, Beijing, China) for 30 minutes at room temperature. Cells were then permeabilized with PBST containing 0.1% Triton X-100 (V/V; P1080, Solarbio, Beijing, China) for 20 minutes at room temperature, and blocked with PBST containing 5% BSA (V/V; A8020, Solarbio, Beijing, China) for 1 hour at room temperature.

Next, the cells were incubated with NF-κB P65 primary antibody (1:100; ab32536, Abcam, Cambridge, MA, USA) overnight at 4°C. After washing with PBST, the cells were incubated with Cy3–conjugated secondary antibody (1:100; SA00009-2, ProteinTech, Wuhan, China) for 1 hour at room temperature away from light. The cells were then washed by PBST again, incubated with DAPI working solution (C0065, Solarbio, Beijing, China) for 10 minutes at room temperature away from light, washed with PBST, and finally added to 1 ml PBS. Images were captured using a laser scanning confocal microscope (NCF950, Nexcope, Ningbo, China).

### 4.10 Statistical analysis

All data are expressed as means ± standard error of the mean (SEM). Statistical significance was tested using GraphPad Prism (Version 10.1.2, GraphPad Software Inc., San Diego, CA, USA). Group means were compared using two-way ANOVA and Tukey’s multiple comparison test. Tukey’s tests were performed only when the F value reached *P* < 0.05 and variance inhomogeneity was not significant. The data represent at least three independent experiments. *P* < 0.05 was considered statistically significant.

## Data Availability

The original contributions presented in the study are included in the article/**Supplementary Material**. Further inquiries can be directed to the corresponding authors.
