# Supplementary material for: Role of LECT2 in exacerbating atopic dermatitis: insight from in vivo and in vitro models via NF-κB signaling pathway
Source: Front Immunol. 2024 Aug 14;15:1439367. doi: 10.3389/fimmu.2024.1439367 (PMC11349537; doi:10.3389/fimmu.2024.1439367)
Supplement: Supplementary file 1 [file Table1.docx]

**Supplementary Data**

**Supplemental Table 1 (Table S1). The RT-PCR primers used in this study (Mouse)**

| Primer | Forward (5’→3’) | Reverse (5’→3’) |
| --- | --- | --- |
| GAPDH | TCGAGTGACAAACACGACTGC | AATGTGTCCGTCGTGGATCT |
| IL-1β | TGGACCTTCCAGGATGAGGACA | GTTCATCTCGGAGCCTGTAGTG |
| IL-4 | ACAGGAGAAGGGACGCCAT | GAAGCCGTACAGACGAGCTCA |
| IL-6 | AGTTGCCTTCTTGGGACTGA | TCCACGATTTCCCAGAGAAC |
| IL-13 | ACCGAAATGTTGATAGCGACAG | ACAATGCTCTGACAAATGCGTA |
| RANTES | GCTGCTTTGCCTACCTCTCC | TCGAGTGACAAACACGACTGC |
| TSLP | CGGATGGGGCTAACTTACA | TCCTCGATTTGCTCGAACTT |
| TNF-α | GGTGCCTATGTCTCAGCCTCTTTT | GCCATAGAACTGATGAGAGGGAG |
| IFN-γ | TCAAGTGGCATAGATGTGGAAGAA | TGGCTCTGCAGGATTTTCATG |
