# Supplementary material for: Role of LECT2 in exacerbating atopic dermatitis: insight from in vivo and in vitro models via NF-κB signaling pathway
Source: Front Immunol. 2024 Aug 14;15:1439367. doi: 10.3389/fimmu.2024.1439367 (PMC11349537; doi:10.3389/fimmu.2024.1439367)
Supplement: Supplementary file 2 [file Table2.docx]

**Supplemental Table 2 (Table S2). The RT-PCR primers used in this study (Human)**

| Primer | Forward (5’→3’) | Reverse (5’→3’) |
| --- | --- | --- |
| GAPDH | CTGGGCTACACTGAGCACC | AAGTGGTCGTTGAGGGCAATG |
| TNF-α | GAGTGACAAGCCTGTAGCCCA | AGCTCCACGCCATTGGC |
| IL-1β | GATATGGAGCAACAAGTGGT | AGGACAGGTACAGATTCTTTTC |
| IL-4 | CACCGAGTTGACCGTAACAGACATC | CGTACTCTGGTTGGCTTCCTTCAC |
| IL-6 | GGTACATCCTCGACGGCATC | GCTCTGGCTTGTTCCTCACT |
| IL-13 | TGTTTGTCACCGTTGGGGAT | TGAGTCTCTGAACCCTTGGC |
| RANTES | CTGCCTCCCCATATTCCTCGG | GAGTTGATGTACTCCCGAACCC |
| TSLP | TATGAGTGGGACCAAAAGTACCG | GGGATTGAAGGTTAGGCTCTGG |
